# Dr. Christie on Blood-Letting in Hydrophobia

**Published:** 1813-02

**Authors:** Thomas Christie

**Affiliations:** Of the Royal College of Physicians, London. Cheltenham


*To the Editors of the Medical and Physical Journal.*

GENTLEMEN,

I HAVE read, with the greatest satisfaction, in your late Numbers, the cases of hydrophobia treated successfully by copious blood-letting, and place great confidence in them, as the reporters, Dr. Berry of Madras, and Dr. Shoolbred of Calcutta, are well known, to almost every practitioner who has been in India, as physicians of very accurate observation and high professional character.

I have been the more pleased with Dr. Shoolbred's interesting case, because it is followed by some very able remarks on the analogy between hydrophobia and tetanus, from which he suggests the probability of the new practice being successfully extended to both diseases, an idea which I confess struck me forcibly when I first perused Dr. Berry's report.

From the great number of cases of hydrophobia Dr. Shoolbred has met with at Calcutta, and my own more limited experience in Ceylon, I am disposed to think that hydrophobia, like tetanus, to which it bears so strong an analogy in other respects, is also a disease of more frequent occurrence in the East Indies, than in the more moderate climate of Europe; and, I confess I entertain sanguine hopes, that, if future experience confirms the efficacy of copious blood-letting in hydrophobia, the same remedy may, with equal advantage, be extended to tetanus.

We have no general hospital at Columbo for the reception of the natives as at Calcutta, and the population is comparatively trifling; but, during my residence at the former place as superintendant of hospitals in Ceylon, I met with three unsuccessful cases of hydrophobia amongst the troops quartered there.

The two first cases, which occurred in a Malay soldier, and a boy belonging to His Majesty's 51st regiment of foot, were easily referred to the bite of a rabid dog; but, in the last, no such cause could be traced, after the most minute inquiry, and it was only from the similarity of symptoms, and fatality of the disease, that such a cause was even suspected.

The patient, a young man of the name of Gorman, belonging to the band of the 19th regiment, could assign no cause for his disease except the having played on a wind instrument on board ship, the third night before his death, when greatly oppressed with sea sickness. This happened on Saturday, in May 1808; and, on the Monday following, he he was received into the Regimental Hospital at Columbo, complaining of difficulty of swallowing. He had also, in the words of my notes taken at the time, "the greatest horror at even the mention of liquids, great anxiety and violent burning sensations at the epigastrium, was constantly throwing out his saliva to a distance, at times outrageous, and at other times composed." He died the next day (Tuesday), notwithstanding the vigorous use of cold affusion, mercury, and opium.

It is chiefly from the analogy between hydrophobia and tetanus, mentioned by Dr. Shoolbred, that I am disposed with him to hope that both diseases may be successfully combated by the same powerful remedy; but it is also worthy of remark, that, in the only successful case of tetanus proceeding from a wound which I ever saw, fourteen ounces of blood were taken from a vein, and afterwards the usual quantity by amputation of the arm, with apparent relief, though my judgment at the time was so warped with the idea of spasm, and dread of debility, that the practice was not followed up; but the patient was afterwards successfully treated with very large doses of opium, and the frequent use of the warm bath, which was generally persevered in for half an hour, till it produced "*faintness* with profuse perspiration,"* an effect not unlike the complete relaxation produced by copious blood-letting.

Your correspondent, Mr. Bampfield, has suggested the trial of copious blood-letting in rabid dogs, and I beg leave to urge the propriety of making the same experiment in horses affected with tetanus, which my professional brethren in the East may have frequent opportunities of doing, as the disease is often produced there after the operation of docking the tail; and I never knew a single instance of recovery in any horse affected with tetanus from this cause.

In answer to the first of the queries for India in your Number for November last, I may observe that Bhang is produced from the Cannabis Sativa of Wildenow, which, in Ceylon, is cultivated in the gardens; and that the dried plant is smoked by the Malay soldiers there, with or without the addition of opium, and produces intoxicating effects similar to that drug.

I do not consider myself sufficiently versed in Eastern languages or literature to venture a reply to the other

---

* For the particulars of this and several other cases of tetanus, treated at Columbo, see Edinburgh Med. Jour. for October, 1812, page 411.

queries, queries; but shall candidly confess, that, having myself never seen, during thirteen years' residence in India, an artificial nose, nor conversed with any person who had, I was disposed to consider the reports of the practice of the Taliocotian art, in that quarter, as either fabulous, or founded on a few rare instances.

THOMAS CHRISTIE, M.D.
Of the Royal College of Physicians, London.

*Cheltenham, Jan.* 4, 1813.

